# Facile Separation of Gadolinium(III) from Samarium(III) and Lanthanum(III) by Emulsion Liquid Membrane and the Optimization with the Box‐Behnken Design Method

**DOI:** 10.1002/open.202500378

**Published:** 2025-10-13

**Authors:** Uji Pratomo, Santhy Wyantuti, Natasha Fransisca, Husein Hernandi Bahti, Retna Putri Fauzia, Ari Hardianto, Husain Akbar Sumeru, Dwi Ratna Setiani, Tiny Agustini, Syulastri Effendi

**Affiliations:** ^1^ Department of Chemistry Universitas Padjadjaran Sumedang West Java 45363 Indonesia; ^2^ National Research and Innovation Agency Research Centre for Environment and Clean Technology Bandung West Java 40135 Indonesia; ^3^ Department of Pharmacy Al‐Ghifari University Bandung Bandung West Java 40293 Indonesia

**Keywords:** Box‐Behnken design, chemometric, emulsion liquid membrane, rare earth elements, separation

## Abstract

Rare earth elements (REEs) are important topics and receive considerable attention, because of their unique properties, high economic value and are widely applied in various fields. Gadolinium is an REE, commonly used as a contrast agent for magnetic resonance imaging. However, its presence is still mixed with other REE like samarium and lanthanum so it's necessary to separate gadolinium from the mixture. The purpose of this research is to separate gadolinium from samarium and lanthanum and to determine the optimum conditions of various parameters that affect their separation. This separation is carried out by emulsion liquid membrane method using tributyl phosphate extractant, span‐80 and tween‐80 surfactants, kerosene solvent and nitric acid as internal and external phases. Parameter optimization is carried out with Box‐Behnken design (BBD) which can predict the optimum value efficiently. The results are analyzed using visible spectrophotometer with alizarin red sulfonate. In this research, gadolinium is successfully separated from samarium and lanthanum with optimum conditions: surfactant concentration 4.2%, ligand concentration 1.4%, internal aqueous concentration 2.3 M, and external aqueous pH 1. The results obtained gadolinium with a value of %E and %S being 84.18% and 89.24%, while the recovery and purity are 75.12% and 22.59%.

## Graphical abstract

Facile separation of Gd(III) from Sm(III) and La(III) is achieved using an emulsion liquid membrane with tributyl phosphate as extractant. Optimization by Box‐Behnken design reveals critical parameter interactions, enabling improved selectivity and efficiency, highlighting the potential of this approach for rare earth element purification.

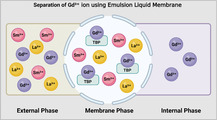

## 1 Introduction

At present, rare earth elements (REEs) are an important and interesting topic, and receive considerable attention^[^ ^1^ ^]^ because of their unique properties, high economic value, and being useful in various fields.^[^ ^2^ ^]^ Some REEs are widely applied in conventional industrial fields such as metallurgy, ceramics, magnets, electronics, nuclear, and widely applied in advanced industries such as hybrid cars, cell phones, neon lights, and defense technology.^[^ ^3^ ^,^ ^4^ ^]^

Due to its vast applications of rare earths, the demand for rare earths with high purity and large quantities is increasing.^[^ ^5^ ^]^ However, REEs are not found in a free state, rather in the form of complex phosphate and carbonate compounds. Therefore, REEs need to be separated from complex compounds, so they can be applied in various fields.^[^ ^6^ ^]^ The similarity of physical and chemical properties of REEs make it difficult to separate them, so an appropriate and efficient method is required for the separation process. Several methods have been used for the separation and purification of REEs, such as crystallization, chemical precipitation, ion exchange, adsorption, and extraction.^[^ ^3^ ^,^ ^7^ ^]^ On an industrial scale, extraction is usually used to purify REEs. However, the extraction process has several drawbacks, including the high consumption of solvents and the need for many extraction steps to obtain high‐purity products.^[^ ^8^ ^]^ In addition, this method is less effective at very low metal ion concentrations.^[^ ^9^ ^,^ ^10^ ^]^ Therefore, solvent extraction was developed into a liquid membrane technology involving a selective liquid membrane phase, where the extraction and stripping processes occur in one step.^[^ ^11^ ^]^ There are three main types of liquid membranes, namely emulsion liquid membranes (ELM), bulk liquid membranes (BLM), and supporting liquid membranes (SLM).^[^ ^12^ ^]^ Of the three types of liquid membranes, ELM is considered more advantageous because it can achieve a much higher mass transfer area, the ability to process various compounds in a relatively short time, relatively low cost and energy consumption, and the emulsion can be reused after undergoing a demulsification process.^[^ ^13^ ^,^ ^14^ ^]^

The advantages of the ELM method led the authors to be interested in separating REEs using this method. The REEs that are separated is gadolinium from samarium and lanthanum. These three elements were taken from different REE groups, namely gadolinium and samarium from the medium group REEs and lanthanum from the light REE groups. These three elements are usually found in monazite sand which is abundant in the Bangka, Belitung, and Singkep Islands.^[^ ^15^ ^]^ At present, many parties are eager to obtain high‐purity gadolinium, because gadolinium is usually used in the medical field as a contrast agent in magnetic resonance imaging (MRI).^[^ ^3^ ^]^ In monazite sand, the presence of gadolinium is still mixed with other rare earths, such as samarium which is in a group position and has an atomic number close to gadolinium and lanthanum which has a high concentration in monazite sand and whose presence interferes with other rare earths. This causes the presence of gadolinium to be considered impure, so it is necessary to separate it from the mixture. In the separation process, the ELM method is influenced by the selectivity of the ligand used. Lanthanide elements can form good chelating compounds with phosphoric acid and tributyl phosphate (TBP). Thus, these ligands can be used to extract lanthanide elements.^[^ ^16^ ^]^ In this study, TBP ligand was selected as the extractant. TBP is an extractant that is quite stable to acids, cheap, easy to obtain, and has a high coefficient of distribution and selectivity, so it is good enough for rare earth separation.^[^ ^1^ ^]^

In the process of separating REEs using the ELM method, there are several parameters that interact with each other and greatly influence the success of the separation process, so it is necessary to carry out the optimization stage to obtain optimal separation results.^[^ ^17^ ^]^ If the test for each parameter is done individually, it will take a long time and cost a lot, causing the research to be less effective.^[^ ^18^ ^]^ Therefore, in this study, an experimental design was used to minimize the number of trials, facilitate the selection of parameters for a significant response, and obtain optimal results.^[^ ^19^ ^]^ In addition, experimental design can be used to improve quality and productivity with a 95% confidence level.

Currently, the experimental design has developed into a response surface methodology (RSM), which is a statistical and mathematical technique that can be used to determine the interaction between parameters to responses and to optimize various parameters simultaneously.^[^ ^18^ ^]^ The determination of the optimum design of a method by RSM is carried out through several stages including screening, improvization, and determining the optimum point. The screening stage was using result of previous experiment^[^ ^20^ ^]^ regarding the selection of parameters that have a significant influence on the separation of REEs by ELM with a two‐level factorial experimental design. As a result, the selected parameters that have a significant influence on the response include the pH of the external phase, the concentration of the internal phase, the concentration of ligands, and the concentration of surfactants. Based on the selected parameter conditions, parameter optimization through RSM needs to be carried out including the Box‐Behnken design (BBD) or central composite design (CCD) methods.^[^ ^21^ ^]^ One of the differences between BBD and CCD is that in the BBD design there is no axial run, resulting in fewer experimental units and more efficient experiments.^[^ ^22^ ^]^ In this study, there are four parameters that need to be optimized, and BBD requires at least three levels for each factor in the experiment.^[^ ^18^ ^]^ Therefore, the BBD method is suitable for this research.

In previous research,^[^ ^15^ ^]^ Th was separated from Ce hydroxide in monazite with TBP. From this research, the extraction efficiency of Ce = 84.54%, the stripping efficiency of Ce = 98.05%, and the total efficiency of Ce = 82.85%, the extraction efficiency of Th = 46.41%, the stripping efficiency of Th = 87.68%, and the total efficiency of Th = 40.69%.^[^ ^15^ ^]^ Meilinda et al. (2021)^[^ ^17^ ^]^ have also separated gadolinium(III) from samarium(III) with TBP and D2EHPA extractants by ELM with extraction efficiency results 72.48% for Gd(III) and 38.36% for Sm(III).^[^ ^17^ ^]^ Yulandra et al. (2020)^[^ ^18^ ^]^ have optimized REEs precipitation from REEs concentrate using the BBD method. From this study, the results obtained for the purity of REEs were 97% and the recovery was 95%.^[^ ^18^ ^]^ These three studies have been confirmed to be successful and provide good separation results. However, in the separation of metal ions, the use of TBP as an extractant is often considered less selective, so many studies have used TBP together with more selective extractants such as D2EHPA. In Indonesia, there is still little research on the separation of REEs by ELM using TBP as the single extractant and its optimization using an experimental design, so this research needs to be conducted to determine the optimum conditions for TBP of REEs separation by ELM.

Based on the description above, this research focuses on separating Gd(III) from Sm(III) and La(III) using TBP as a single extractant in the ELM system, combined with BBD optimization, which can effectively separate the gadolinium under carefully optimized conditions. Moreover, the application of BBD allowed us to identify the critical interactions among surfactant concentration, ligand concentration, internal phase concentration, and external phase pH, achieving significantly higher extraction and stripping efficiencies compared to earlier reports. To evaluate the success of separation using the ELM method, analysis was performed using a visible spectrophotometer with alizarin red sulfonate (ARS) complexing ligand. This systematic study that integrates ELM–TBP with RSM‐based optimization will address the practical challenge of gadolinium separation from a ternary REE mixture, making the approach more efficient, economical, and scalable.

## 2 Experimental Section

### 2.1 Materials and Apparatus

The chemicals used in this study are distilled water, ARS (Sigma‐Aldrich), acetic acid (Merck, pa), nitric acid (Merck, pa), gadolinium oxide (Sigma‐Aldrich 99.9%), kerosene, lanthanum oxide (Sigma‐Aldrich 99.9%), sodium acetate (Merck 99.9%), sodium hydroxide (Merck 99.9%), samarium oxide (Sigma‐Aldrich 99.9%), span‐80 (Merck), TBP (Sigma‐Aldrich 97%), and tween‐80 (Merck).

The equipment used are pyrex glassware, Eppendorf micropipette, Mettler Toledo analytical balance: AL‐204, Jenwey 3510 pH meter, heater, magnetic stirrer Heidolph MR Hei‐Standard, Uv–vis spectrophotometer, and the IKA‐T25 ultraturrax.

### 2.2 Emulsion Preparation and Extraction of Gd(III), Sm(III), and La(III) by ELM

Span‐80 concentration variation 4, 4.5, and 5% (v/v) mixed with TBP ligand with various concentrations of 0.5, 1, and 1.5% (v/v). Each reagent was put into a different volumetric flask, then dissolved with kerosene up to 40 mL. The various mixtures were transferred to a beaker and stirred with a magnetic stirrer until homogeneous. Then added nitric acid with various concentrations of 1.5, 2, and 2.5 M as much as 40 mL as the internal acid phase slowly while stirring with ultraturrax with a speed variation of 9000 rpm for 15 min until a white emulsion is formed.

For the extraction section, pipette 0.8, 1.6, and 6.4 mL of 1000 ppm of Gd(III), Sm(III), and La(III) solutions were used, respectively, then put into a beaker and diluted with distilled water to 40 mL. This ratio was applied to reproduce a representative distribution in simulated leachates, ensuring that the separation study reflects real ore‐derived feed conditions. Then the pH of the external phase solution was adjusted to pH 1, 2, or 3 using HNO_3_ solution, and then diluted again with distilled water to 80 mL. Then the liquid emulsion solution was added to the beaker, stirred using a magnetic stirrer at 500 rpm for 10 min. The (Gd(III), Sm(III), and La(III))‐(emulsion) solutions were transferred into a separatory funnel and waited for two phases to form, namely the external aqueous phase at the bottom and the membrane phase at the top. After the two phases were formed, the external phase was separated from the membrane phase and its volume was measured, then the membrane phase was waited again until complete demulsification occured and two phases were formed, the internal phase at the bottom and the membrane phase at the top. The two phases were separated again and their volumes were measured.

### 2.3 Determination of the Maximum Absorption Wavelength of ARS and REE–ARS Complexes

As much as 0.5 mL of ARS 0.7% solution was put into a beaker, then the pH of the solution was adjusted to pH 4.0 with the addition of sodium hydroxide solution. Then transferred to a 25 mL volumetric flask and acetate buffer solution of pH 4.0 was added to the mark. The maximum absorption of ARS was measured with a visible spectrophotometer at a wavelength of 400–800 nm. The maximum absorption absorbance is recorded as *λ*max ARS. To determine the *λ*max of the Gd‐ARS, Sm‐ARS, and La‐ARS complex, the same procedure was carried out, but it was necessary to add stock solutions of Gd(III), Sm(III), and La(III) at the beginning of the procedure.

### 2.4 Stability and Calibration of REE–ARS Complexes

In the stability measurement, 100 ppm Gd(III), Sm(III), and La(III) stock solutions were pipetted, 2.5 mL each into a different beaker, the pH of the solution was adjusted to pH 4.0 with the addition of sodium hydroxide. Then transferred to a 25 mL volumetric flask and 0.5 mL of 0.7% ARS solution was added. Added acetate buffer pH 4.0 up to the boundary mark. The maximum absorption was measured using a visible spectrophotometer at *λ*max of the Gd‐ARS, Sm‐ARS, and La‐ARS complexes every 10 min for 1 h to obtain the stability of the Gd‐ARS, Sm‐ARS, and La‐ARS complexes. The blank solution was carried out in the same way, but without the addition of the REE solution.

For the calibration curve, Gd(III), Sm(III), and La(III) solutions were pipetted each with a concentration of 10, 15, 20, 25, and 30 ppm. Then it is placed in a different beaker and the pH is adjusted by adding sodium hydroxide solution until it reaches a pH of 4. The solution is put into a 25 mL volumetric flask, then 0.5 mL of ARS solution is added and distilled water is added up to the mark. Blanks were made in the same way but without adding the metal solution being analyzed. Each REE–ARS complex compound against the blank was analyzed by visible spectrophotometer using the maximum absorption wavelength of each element. The absorbance of each element concentration is plotted on a graph so that a standard curve for each element is obtained.

### 2.5 Verification of Analysis Method

The results of the standard curve are calculated to determine the detection limit (LOD) and quantization limit (LOQ). Furthermore, measurements of Gd‐ARS, Sm‐ARS, and La‐ARS were carried out repeatedly for seven repetitions to obtain accuracy and precision values.

### 2.6 Analysis of Separation Results and Optimization Using BBD

The extract solution in the internal phase and the raffinate solution in the external phase were each diluted in a pipette of 10 mL into a different beaker and the pH of the solution was adjusted to a pH of 4.0 with the addition of sodium hydroxide solution. Then transferred to a 50 mL volumetric flask and added 1 mL of 0.7% ARS solution, and diluted with acetate buffer pH 4.0 to the mark. The blank solution was carried out in the same way without the addition of the REE solution being analyzed. The absorbance of each of the Gd‐ARS, Sm‐ARS, and La–ARS complex solutions to the blank was measured with a visible spectrophotometer at the maximum absorption wavelengths of Gd‐ARS, Sm‐ARS, and La‐ARS to obtain *λ*max Gd(III), Sm(III), and La(III). Then calculations were performed to obtain the concentrations of Gd(III), Sm(III), and La(III) contained in the extract solution (internal phase) and raffinate solution (external phase).

Optimization parameters of separation of Gd(III) from Sm(III) and La(III) were carried out with the BBD experimental design. There are four parameters that significantly influence the separation of Gd(III) from Sm(III) and La(III), including surfactant concentration, ligand concentration, internal phase concentration, and external phase pH as listed in **Table** **1**.

**Table 1:** Selected parameters optimized with the BBD response surface method.

Parameter	Code	Level		
		−1	0	+ 1
Surfactant concentration [%]	A	4	4.5	5
Ligand concentration [%]	B	0.5	1	1.5
Internal phase concentration (M)	C	1.5	2	2.5
External phase pH	D	1	2	3

## 3 Results and Discussion

### 3.1 Separation of Gd(III) from Sm(III) and La(III) by ELM

**Figure 1:**
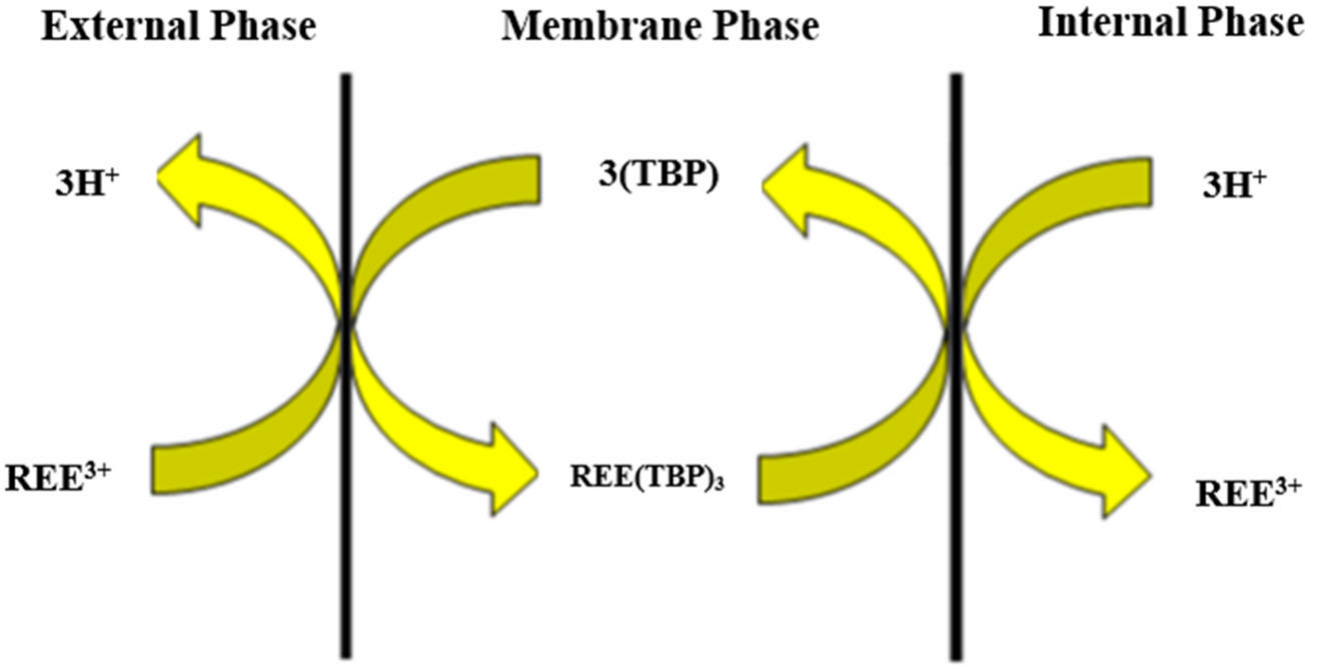
Mechanism of separation of REE by ELM (adopted from research by Davoodi‐Nasab et al. (2018)^[^ ^23^ ^]^).

In this research, the optimum conditions for the separation of Gd(III) from Sm(III) and La(III) were determined using TBP ligands by ELM through the BBD experimental design, with kerosene as an organic solvent, span‐80 and tween‐80 as surfactants, and nitric acid as the internal phase. The ELM separation involves three phases, namely the external phase (feed), the membrane phase, and the internal phase. The feed phase is often referred to as the external water phase in which there are metal ions, Gd(III), Sm(III), and La(III) ions, to be separated. The membrane phase is an organic phase in which there are TBP extractants and span‐80 and tween‐80 surfactants in kerosene organic diluent. The internal phase is the aqueous phase in which there are stripping agents such as nitric acid. The mechanism for separating REEs by ELM is shown in **Figure** **1**.^[^ ^23^ ^]^

In addition to separation efficiency, emulsion stability was also observed, since breakage and phase inversion are known challenges in ELM systems. Under the optimized conditions, the emulsions remained stable during the 10 min extraction process and subsequent demulsification, with no visible leakage or premature breakage. The reproducibility of emulsion performance was confirmed by triplicate runs, which yielded consistent extraction efficiencies with relative standard deviations (RSD) below 2%. These results indicate that the separation performance reported in this study was achieved under stable and reproducible emulsion conditions.

During extraction, REE ions in the external phase diffuse into the membrane to bind to TBP as a ligand on the outer surface of the membrane phase, resulting in a complex between REE and TBP that dissolves in the membrane phase as follows^[^ ^15^ ^]^

(1) $${\mathrm{REE}}_{\left(\mathrm{aq}\right)}^{3+}+{3(\mathrm{TBP})}_{\left(\mathrm{org}\right)}\;⇄\;{\mathrm{REE}(\mathrm{TBP})}_{3(\mathrm{org})}$$

**Figure 2:**
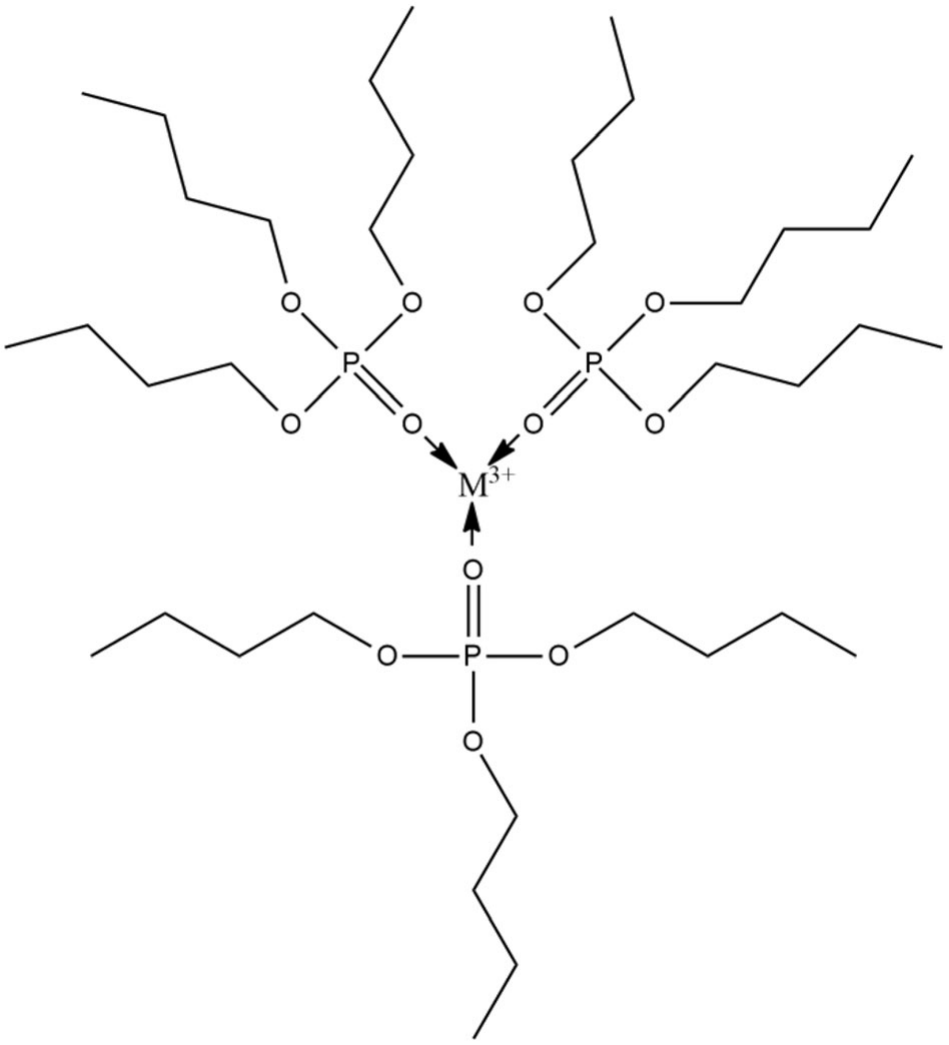
Predicted structure of the REE complex with TBP.

Furthermore, this complex diffuses into the membrane phase on the surface of the membrane phase, then the REE ions are released in the internal phase and replaced with hydrogen ions. Release of REEs is due to ion transport from the external phase driven by the concentration gradient between the external phase and the internal phase. The structure of the REE complex with TBP is not known with certainty, while the assumptions are as follows **Figure** **2**.

### 3.2 Analysis of Separation Results of Gd(III) from Sm(III) and La(III) with Visible Spectrophotometer

#### 3.2.1 Maximum Absorption Wavelengths of Alizarin Red Sulfonate (ARS) and Gd‐ARS, Sm‐ARS, La‐ARS Complexes

**Figure 3:**
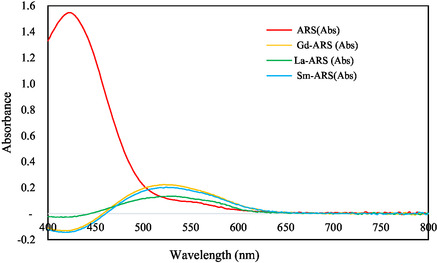
Maximum wavelength curves of ARS, Gd(III)‐ARS, Sm(III)‐ARS, and La(III)‐ARS complexes.

The maximum wavelength of ARS and REE–ARS was determined using a visible spectrophotometer, namely at a wavelength of 400–800 nm. It is necessary to determine the maximum wavelength, because measurements at the maximum wavelength have a higher sensitivity than the others, so that errors in measurement can be minimized. In determining the maximum wavelength, each REE must be complexed with a dye. In this study, (ARS was used which can form water‐soluble complexes with many elemental ions, especially for the determination of Al^3+^, Ga^3+^, Ln^3+^, and other REEs. Gd(III), Sm(III), and La(III) will form red‐purple complexes with ARS at pH 4–5, so it is necessary to add acetate buffer to maintain the pH range between 4 and 5. The maximum wavelength results of Gd‐ARS, Sm‐ARS, and La‐ARS are shown in **Figure** **3**.

As shown in Figure 3, the maximum absorption wavelength for ARS is 422 nm, for the Gd‐ARS complex is 522 nm, Sm‐ARS is 524 nm, and La‐ARS is 529 nm. It is observed that there was a shift in the maximum wavelength of ARS from 422 nm to 522, 524, and 529 nm after the addition of REE solution. This indicates the occurrence of complex formation between Gd(III), Sm(III), and La(III) with ARS. This wavelength shift occurs in the greater direction and is referred to as a bathochromic shift. This is due to the conjugation extension (*π* electron delocalization) of the compound structure, so the transition energy from n → π* will be smaller and the wavelength will be larger.^[^ ^24^ ^,^ ^25^ ^]^

#### 3.2.2 Complex Stability of Gd‐ARS, Sm‐ARS, and La‐ARS

**Figure 4:**
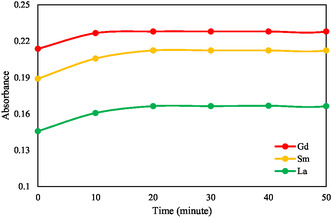
Complex stability curve of Gd(III)‐ARS, Sm(III)‐ARS, and La(III)‐ARS.

The measurement of REE–ARS complex stability was carried out to determine the formation time of the complex and the stability time of the REE–ARS complex. REE will form a colored complex when reacted with ARS. This colored complex can absorb light in the visible light region. Measurement of the stability of the REE–ARS complex was carried out every 10 min and Within 50 min by making a relationship curve between the absorbance value and time as shown in **Figure** **4**.

From Figure 4, it can be seen that the absorption intensity of REE–ARS was formed in the first 20 min and was stable for up to 50 min. This shows that the REE–ARS complex is stable within 20–50 min, which shows that the REE measurements can be made after ARS is added and the solution is allowed to stand for at least 20 min.

#### 3.2.3 Standard Curves of Gd‐ARS, Sm‐ARS, and La‐ARS

**Figure 5:**
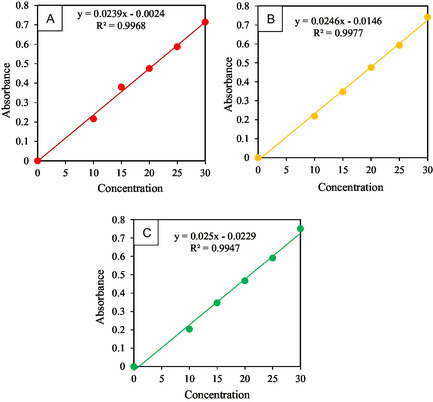
Standard curves of: a) Gd(III)‐ARS; b) Sm(III)‐ARS; and c) La(III)‐ARS.

The Gd‐ARS, Sm‐ARS, and La‐ARS standard curves aims to see the linearity of the absorbance, where this curve can describe the relationship between absorbance and standard solution concentration in various concentration ranges. The preparation of this standard curve was carried out with various concentrations of 10, 15, 20, 25, and 30 ppm at *λ*max 522, 524, and 529 nm for Gd‐ARS, Sm‐ARS, and La‐ARS. The standard curve is shown in **Figure** **5**.

Standard curve linearity can describe the accuracy of the analysis of a method indicated by the value of the correlation coefficient. From the results of the standard curve in Figure 5, the correlation coefficient (R^2^) for the Gd‐ARS complex was 0.9968, Sm‐ARS was 0.9977, and La‐ARS was 0.9947. The value of this correlation coefficient indicates a linear relationship between concentration and the resulting absorption. This is in accordance with Lambert Beer's law which states that there is a linear relationship between the absorbance value and the concentration of the solution.^[^ ^26^ ^]^ Based on the results of R^2^ obtained, the correlation coefficient gives linear results because it meets acceptable criteria, namely 0.9.^[^ ^22^ ^]^

#### 3.2.4 Verification of Analysis Method

The results of the standard curve can be used to verify the analytical method by finding the values for accuracy, precision, recovery, LOD, and LOQ. The data obtained from the concentration of each analyte that gives a different absorbance is processed to determine the LOD and LOQ. LOD test results for Gd‐ARS, Sm‐ARS, and La‐ARS of 1.809, 1.529, and 2.464 ppm, meaning the lowest limit of Gd‐ARS, Sm‐ARS, and La‐ARS for analysis using the verified method is 1.809, 1.529, and 2.464 ppm. Meanwhile, the LOQ test results for Gd‐ARS, Sm‐ARS, and La‐ARS of 5.481, 4.634, and 7.040 ppm, meaning that the concentration is the lowest amount of the sample that can still be determined and meets the agreed accuracy and precision criteria. From these results, it can be interpreted that the concentration used in making the standard curve can still be detected by the instrument and still meets the test criteria.

Furthermore, measurements were repeated seven times to determine accuracy and precision. In this study, the accuracy for Gd‐ARS was obtained; Sm‐ARS and La‐ARS of 99.95%, 99.97%, and 97.95% with a% error of 0.045%, 0.030%, and 2.055%. The recovery value (%recovery) from the analysis results for Gd‐ARS, Sm‐ARS, and La‐ARS were 99.5%, 99.97%, and 97.95%. Based on the literature, these results are acceptable because they meet the requirements, namely the accuracy and % recovery values are in the range of 90%–107%.^[^ ^27^ ^]^

Precision can be determined based on the RSD, which is the ratio of the standard deviation to the average of the test results. The method is declared to have a high level of precision if the %RSD value resulting from the analysis is less than 2/3 (CVHorwitz).^[^ ^27^ ^]^ From the results of the analysis, the %RSD values for Gd(III), Sm(III), and La(III) were 0.16%, 0.13%, and 0.18% and the yield of 2/3 (CVHorwitz) for Gd(III), Sm(III), and La(III) is 0.849, 0.849, and 0.852. The %RSD value of the analysis results is smaller than the 2/3 value (CVHorwitz). Thus, it can be concluded that the verified method has a high level of precision.

### 3.3 Analysis of the Optimization Results of Separation of Gd(III) from Sm(III) and La(III) with the BBD Method

Separation of Gd(III) from Sm(III) and La(III) by ELM is influenced by several parameters that interact with each other, so it is necessary to carry out the parameter selection and optimization stages to obtain optimum separation results. To make it easier to determine the optimum value and to save costs and time for the experiment, optimization is carried out with the help of experimental design through the RSM. In determining the optimum point through RSM, a screening stage or parameter selection is required first. Parameter selection has been carried out by Effendi et al.^[^ ^20^ ^]^ in a previous study, which aims to select parameters that have a significant influence on the separation of REEs by ELM, namely using the help of a two‐level factorial design. Based on the selection results, the parameters of surfactant concentration, ligand concentration, internal aqueous phase acid concentration, and external aqueous phase pH were stated to have a significant effect on the response. So, these results are followed by the parameter optimization stage using BBD (**Table** **2**) to optimize the selected parameters in Gd(III) from Sm(III) and La(III) separation by ELM. The four parameters selected using the BBD were optimized to produce 29 experimental variations.

**Table 2:** Matrix of BBD runs to optimize separation of Gd(III) from Sm(III) and La(III) by ELM.

Surfactant concentration [%]	Ligand concentration [%]	Internal phase concentration (M)	External phase pH	Gd (ppm)	Sm (ppm)	La (ppm)
5	1	2	1	6.70	10.13	17.80
4.5	1.5	2	3	8.35	10.26	17.36
4.5	1	2	2	8.50	10.95	20.74
4.5	0.5	1.5	2	7.92	11.93	15.10
4.5	1	2.5	3	7.45	11.14	28.26
4.5	1	2	2	9.02	10.18	17.27
4.5	1	2	2	8.58	10.06	19.95
4.5	1.5	2	1	7.97	9.55	16.10
4.5	1	1.5	1	7.07	12.03	20.86
4.5	0.5	2	1	6.90	10.16	15.75
4	1	2	1	7.02	11.10	17.23
4.5	1	2.5	1	6.62	11.50	16.32
4.5	1.5	2.5	2	8.08	10.16	22.02
5	0.5	2	2	5.75	10.97	15.44
4	1.5	2	2	6.58	9.08	17.78
4.5	0.5	2	3	7.74	12.80	18.49
4.5	1.5	1.5	2	6.27	10.92	15.00
4	1	2.5	2	7.04	9.09	20.13
4	1	2	3	7.81	10.55	19.88
5	1	1.5	2	7.00	10.54	15.51
4	1	1.5	2	7.62	13.92	16.04
4.5	1	2	2	8.58	10.02	16.46
5	1	2.5	2	5.64	14.58	21.16
4	0.5	2	2	6.15	15.03	15.64
4.5	0.5	2.5	2	5.02	11.95	18.36
5	1.5	2	2	6.07	12.73	16.06
5	1	2	3	8.23	12.52	20.01
4.5	1	1.5	3	8.98	11.97	15.75
4.5	1	2	2	8.43	9.08	20.30

Experimental data were entered into the design as a response, then processed with Design‐Expert 10.0.1 software, and the results were analyzed. The analysis carried out includes analysis of variance (ANOVA) which aims to see the suitability between the predicted value and the actual value. Before carrying out the ANOVA test, it is necessary to determine the degree of significance (*α*) that will be used. In this study, *α *= 0.05 was used, meaning that the greatest chance of error that can be formed is 5%. The *p*‐value states that the function of the test results model to test the model components must be *α *< 0.05.

**Figure 6:**
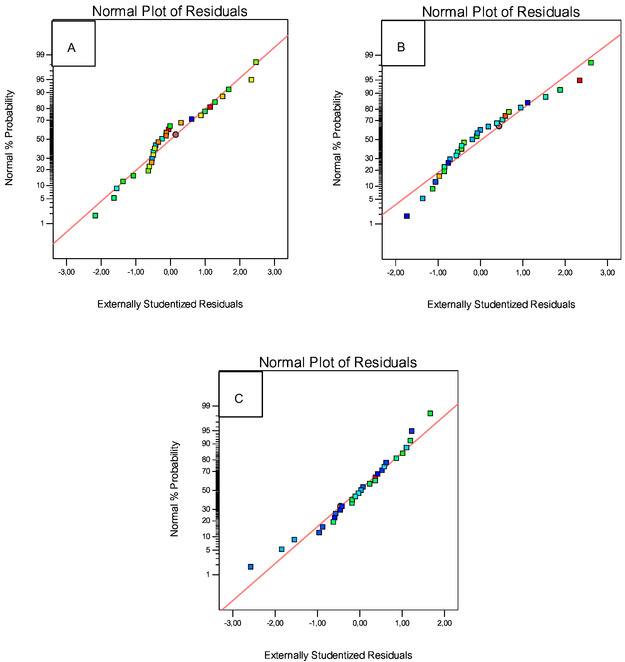
Graph of normal plot of residuals on a) Gd(III); b) Sm(III); and c) La(III) responses.

**Figure 7:**
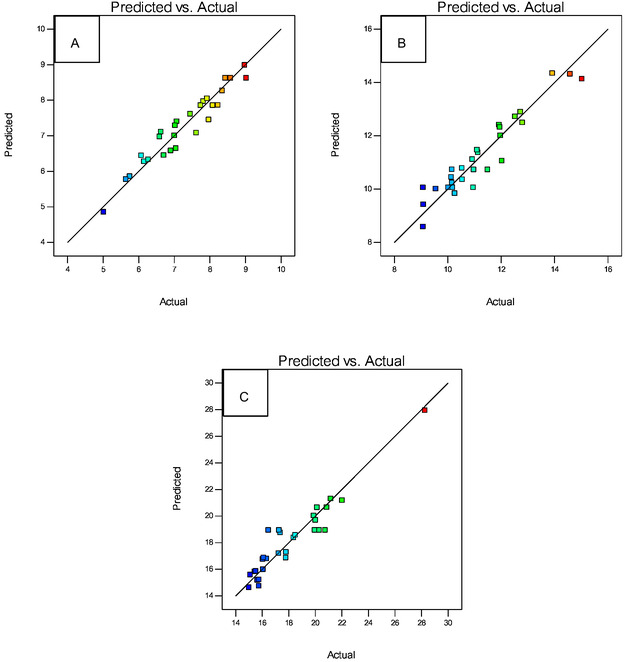
Predicted versus actual graphs of: a) Gd(III); b) Sm(III); and c) La(III) responses.

In addition to ANOVA, in optimizing the separation of Gd(III) from Sm(III) and La(III) using BBD, normality graphs are also obtained as shown in **Figure** **6** and prediction versus actual graphs as shown in **Figure** **7**. These two graphs serve to see the normality of the residual distribution of the 29 experimental variations and to see the suitability between the experimental data and the data produced by the design.

The normal graph of the plot of residuals produced by the design shown in Figure 6 indicates residual values that are close to the normal line. Based on the figure, it can be seen that not all of the experimental response points are on the center line, but they are still spread along the center line between the percentage probability of normality and residuals. Data points that are closer to the normal line indicate that the data is normally distributed.

The normally distributed data indicates that the actual results are close to those predicted by the program. The experimental data values spread around the line which indicates that there is a match between the model and the experimental data. The closer to the normal line, the actual response results will be closer to the predicted response results, as shown in Figure 7.

**Figure 8:**
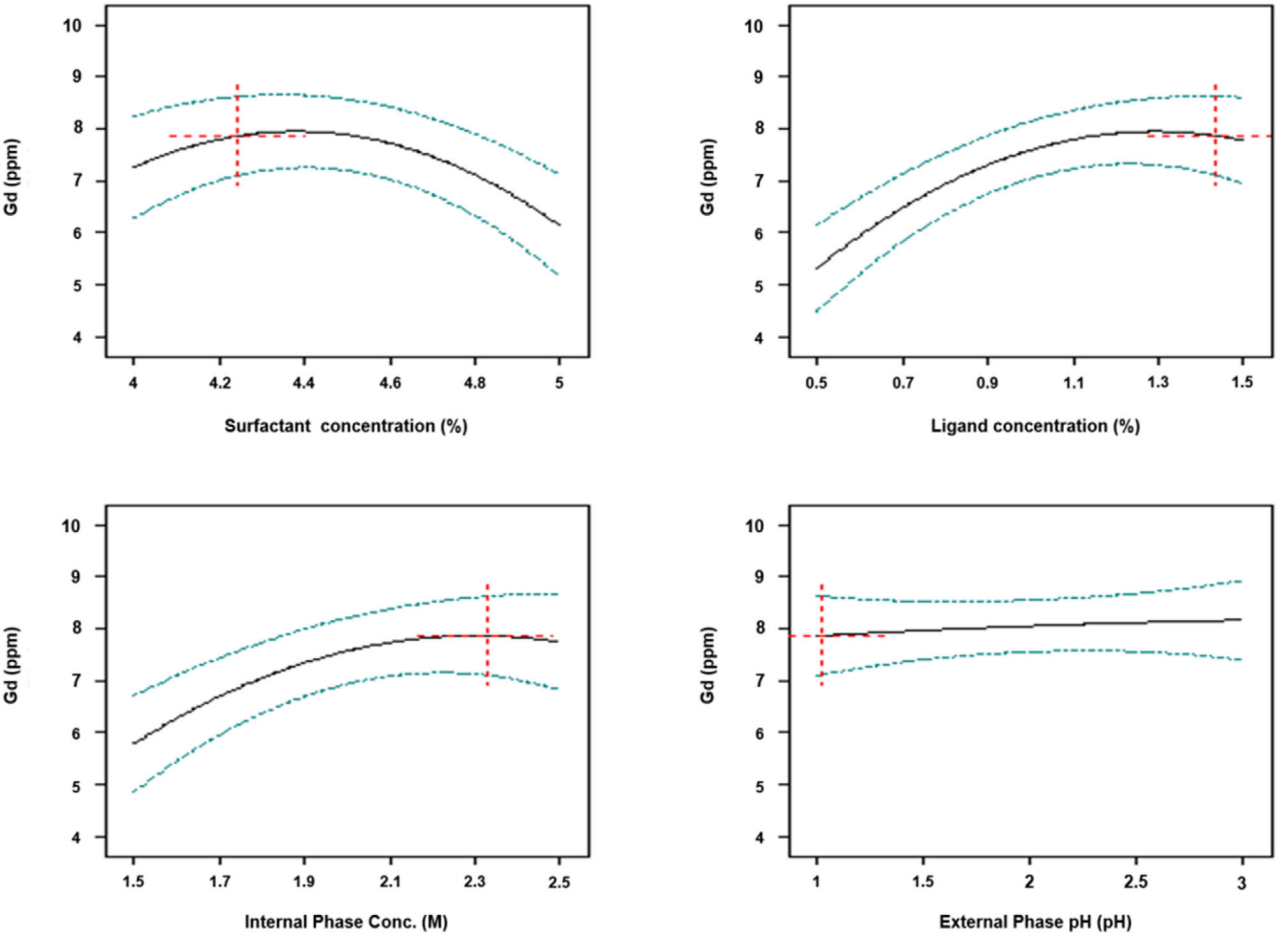
Graph of the interaction of each parameter on the response of Gd(III) concentration.

Optimum condition verification is carried out to test the accuracy of the model produced by the design with the experimental data. The method is by looking at the value of the coefficient of determination (R^2^) from the linear regression curve. If the value of the coefficient of determination (R^2^) is getting closer to 1, then the suitability of a model with the experimental value or the actual value is getting higher. The coefficient of determination resulting from the design was 0.9324 for the response to Gd(III) concentration, 0.9047 for the response to Sm(III) concentration, and 0.9015 for the response to La(III) concentration. This indicates there is a match between the predicted value and the actual value. The process of parameter optimization using BBD also produces a graph of the interaction of each parameter to the response as shown in **Figure** **8**.

Surfactant concentration, ligand concentration, internal phase concentration, and external phase pH had a significant effect on separation. This can be seen from the graph of the relationship between parameters to the response of Gd(III) concentration in Figure 8. It tends to give a line that increases with increasing surfactant, ligand, and internal phase concentrations, and the line will decrease again when it reaches its optimum point. Meanwhile, the pH of the external phase increased/decreased, but not drastically. Therefore, these four parameters have a significant effect on the separation of Gd(III) from Sm(III) and La(III) because the response parameter relationship lines are not horizontal.

**Figure 9:**
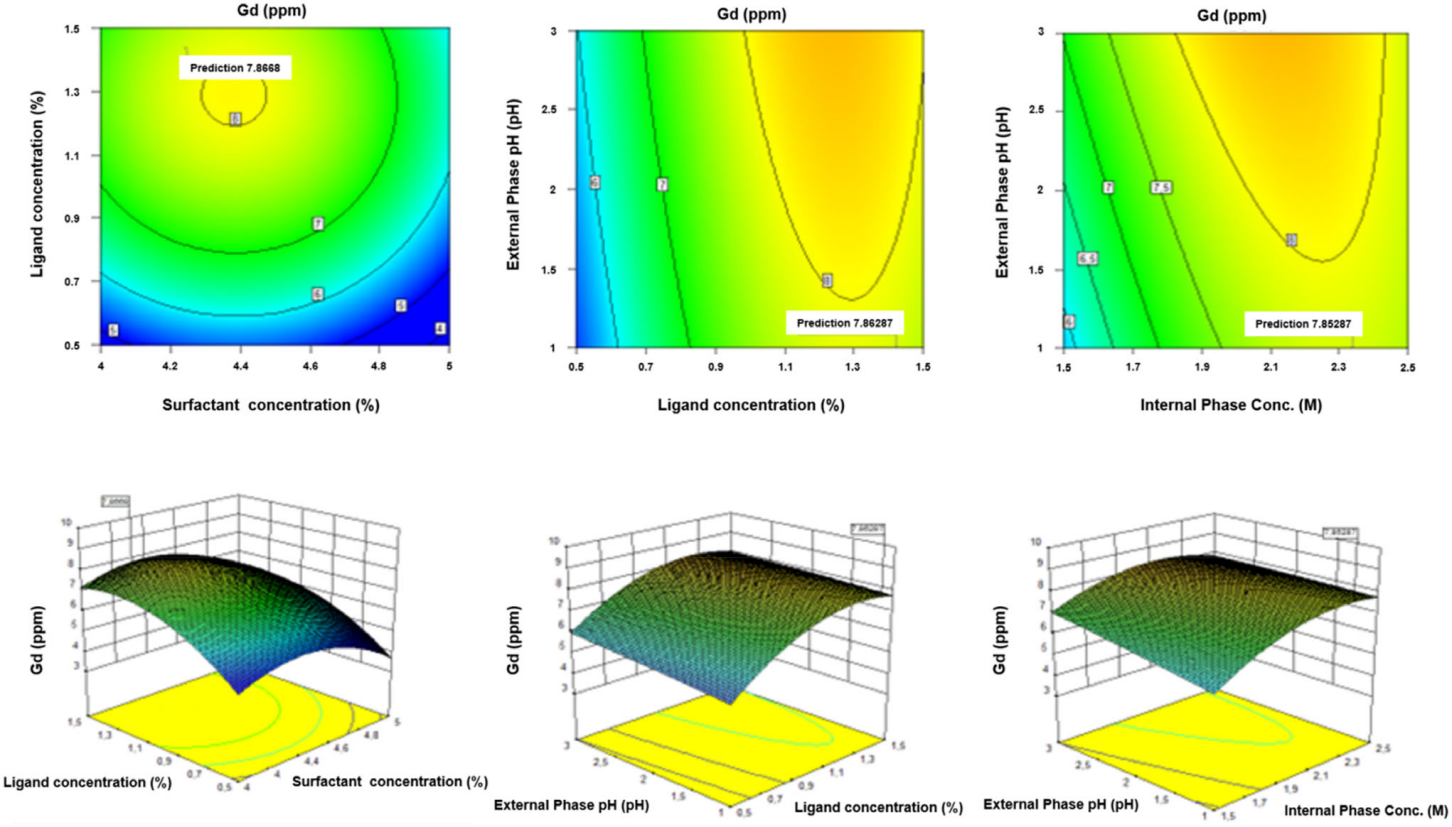
Contour plot (top) and 3D surface (bottom) graphs of response to Gd(III) concentration.

In addition to the graph above, to see the influence between parameters can also be seen in two dimensions through a contour plot and in three dimensions through a surface 3D graph. This graph can be used to determine the optimal level of each variable on the response and to understand the interaction effect of the variables on the response. The graph is shown in **Figure** **9**.

Based on the graph in Figure 9, it can be seen that there is an interaction between the concentration of the ligand and the concentration of the surfactant, the pH of the external aqueous phase and the concentration of the ligand, and the pH of the external aqueous phase and the concentration of the internal aqueous phase on the response of Gd(III) concentration.

The surfactant concentration is directly proportional to the size of the emulsion grains which depends on the viscosity, and the viscosity is directly proportional to the concentration of the ligand, so there is a relationship between the two. Excess concentration of surfactant in the membrane phase will increase the viscosity of the emulsion, which can cause swelling of the emulsion. Likewise, higher ligand concentration will cause the emulsion viscosity to increase and reduce the complex diffusion process in the membrane phase, so the efficiency of separating Gd(III) from Sm(III) and La(III) will decrease.

Ligand concentration and pH of the external phase directly affect extraction and membrane stability. The extraction efficiency is highly dependent on the acidity of the external phase solution. Extraction occurs through the replacement of hydrogen ions in the ligands. The difference in hydrogen ion concentration between the external phase and the internal phase is the driving force for the transport of REEs which will increase as the pH of the external phase increases. This could be due to the rate of complexation of REEs with ligands as cation exchangers in direct proportion to the acidic properties of the external aqueous phase.^[^ ^23^ ^]^

The acid concentration of the internal aqueous phase and the pH of the external aqueous phase affect the separation, where these two phases were prepared under acidic conditions using HNO_3_. The acidic conditions between the external and internal phases must have a great span so that the driving force in Gd(III) extraction increases. The acidity of the internal aqueous phase must be higher than the external aqueous phase, the aim is that there are far more H^+^ ions in the internal aqueous phase, so when the metal ion exchange process occurs from the external to the internal phase, more H^+^ ions enter the external aqueous phase, so the more metal ions that enter the internal phase. Gd(III) concentration increases along with increased acidity, but higher optimization condition will cause a decrease in Gd(III) concentration. If the acidity of the external aqueous phase is too high, it will cause a decrease in extraction efficiency. This can be caused by surfactant damage at lower pH values, thus causing emulsion destabilization. If the acid concentration of the internal phase is too high, the membrane phase covering the internal phase grains will become thinner, causing the emulsion to break more easily due to osmotic pressure and the stripping efficiency decreases.

**Figure 10:**
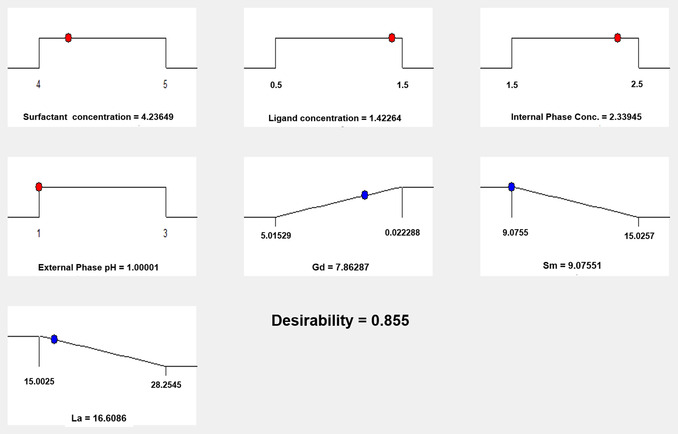
Optimization value of separation of Gd(III) from Sm(III) and La(III) byELM with BBD experimental design.

The measurement responses were processed to obtain the optimum value for each parameter based on the desired response, namely the maximum Gd(III) concentration and the minimum Sm(III) and La(III) concentration in the internal phase. The approach that is more often used for optimization is the desirability function approach by transforming each response to the desired value and denoted by di (desirability) where 0 ≤ di ≤ 1. The function of the desirability approach is to optimize more than one response simultaneously.^[^ ^22^ ^]^ The desirability value range is from 0 to 1 where the desirability value is getting closer to 1, indicating that the program's ability to produce the desired product is more perfect. The objective of optimization is not to obtain a desired value of 1, but to find the best conditions that bring together all objective functions. The results are listed in **Figure** **10**.

### 3.4 Determination of Extraction Efficiency (%E), Stripping Efficiency (%S), Distribution Coefficient, Separation Factor (α), Recovery, and Purity Gd(III) from Sm(III) and La(III)

**Figure 11:**
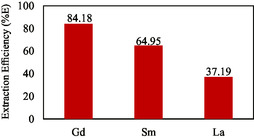
Extraction efficiency of Gd(III), Sm(III), and La(III).

Extraction efficiency (%E) can be determined by calculating the difference between the concentrations of Gd(III), Sm(III), La(III) after extraction and the concentrations of Gd(III), Sm(III), and La(III) in the water phase before extraction. The amount of metal ions extracted into the organic phase is expressed in extraction efficiency (%E). Based on the data obtained, %E Gd(III) forms more complexes than Sm(III) and La(III), with %E Gd(III) of 84.18%, %E Sm(III) of 64.95%, and %E La(III) of 37.19% as shown in **Table** **3** and **Figure** **11**.

**Table 3:** Extraction efficiency of Gd(III) from Sm(III) and La(III) with TBP ligand by ELM.

REE	Initial concentration (ppm)	Internal phase concentration (ppm)	External phase concentration (ppm)	Organic phase concentration (ppm)	%E
Gd(III)	10	7.51	1.58	8.42	84.18%
Sm(III)	20	9.12	7.01	12.99	64.95%
La(III)	80	16.63	50.25	29.75	37.19%

**Figure 12:**
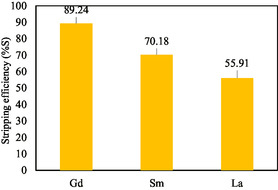
Stripping efficiency of Gd(III), Sm(III), and La(III).

The strength of concentrated acid to remove complex bonds between metals and ligands is expressed in stripping efficiency (%S). Based on the data obtained, %S Gd(III) was 89.24%, %S Sm(III) was 70.18%, and %S La(III) was 55.91%, as shown in **Table** **4** and **Figure** **12**.

**Table 4:** Stripping efficiency of Gd(III) from Sm(III) and La(III) with TBP ligand by ELM.

REE	Initial concentration (ppm)	Internal phase concentration (ppm)	External phase concentration (ppm)	Organic phase concentration (ppm)	%S
Gd(III)	10	7.51	1.58	8.42	89.24%
Sm(III)	20	9.12	7.01	12.99	70.18%
La(III)	80	16.63	50.25	29.75	55.91%

**Figure 13:**
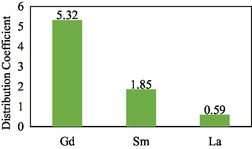
Distribution coefficient of Gd(III), Sm(III), and La(III).

The value of the distribution coefficient can be determined by comparing the concentration extracted into the organic phase with the concentration remaining in the external aqueous phase. The resulting distribution coefficient in the separation of Gd(III) from Sm(III) and La(III) with the TBP ligand was Gd = 5.32, Sm = 1.85, and La = 0.59 as shown in **Table** **5** and **Figure** **13**.

**Table 5:** Distribution coefficient of Gd(III) from Sm(III) and La(III) with TBP ligand by ELM.

REE	Initial concentration (ppm)	Internal phase concentration (ppm)	External phase concentration (ppm)	Organic phase concentration (ppm)	Distribution coefficient
Gd(III)	10	7.51	1.58	8.42	5.32
Sm(III)	20	9.12	7.01	12.99	1.85
La(III)	80	16.63	50.25	29.75	0.59

The distribution coefficient values obtained from previous calculations are used to determine the separation factor (*α*) of Gd(III), Sm(III), and La(III). Determination of the separation factor (*α*) was carried out to see the separating power of Gd(III), Sm(III), and La(III). The separation factor value must be *α *> 1 which is a requirement for a metal to be separated from the mixture. The separation factor values obtained include *α*Gd‐Sm is 2.87, *α*Gd‐La is 8.99, and *α*Sm‐La is 3.13. This shows that in mixed systems, the metals Gd(III), Sm(III), and La(III) with TBP ligands can be separated. The greater the separation factor, the easier to separate metal mixtures.

**Figure 14:**
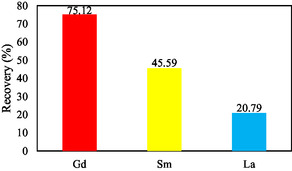
Recovery of Gd(III), Sm(III), and La(III).

**Figure 15:**
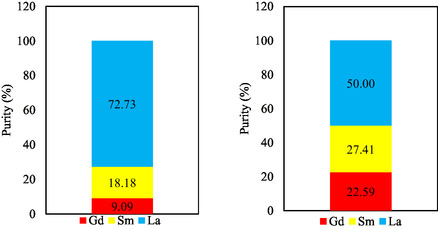
Purity of Gd(III), Sm(III), and La(III); initial (left) and final (right).

Based on the results obtained, the recovery and purity of Gd(III) from Sm(III) and La(III) were determined. From these calculations, the recovery of Gd(III) is 75.12%, Sm(III) is 45.58%, and La(III) is 20.79% as shown in **Figure** **14** with a purity of Gd(III) 22.59%, Sm(III) 27.41%, and La(III) 50.00% as shown in **Figure** **15**.

From Figure 15 it can be seen that the purity of Gd(III) is lower than Sm(III) and La(III), because the initial concentration of Gd(III) in the mixture with Sm(III) and La(III) is only 9.09%. However, the purity has increased from initially 9.09% to 22.59%. When viewed from the recovery, gadolinium has the greatest value than the other REEs. This can also be seen from the %E and distribution coefficient values of Gd(III) which show the highest values, meaning that Gd(III) has a higher ability to extract than Sm(III) and La(III).

Although the recovery of Gd(III) (75.12%) is not close to quantitative, it remains substantially higher than that of Sm(III) (45.58%) and La(III) (20.79%). More importantly, the separation factors (*α*Gd‐Sm = 2.87; *α*Gd‐La = 8.99) confirm that gadolinium was preferentially extracted relative to samarium and lanthanum under the optimized conditions. This indicates that the selectivity of the method is better reflected by the separation factor rather than the absolute recovery values, which are also influenced by emulsion stability and diffusion resistance inherent to the ELM process. Thus, while the method does not achieve complete recovery, it demonstrates meaningful selectivity for Gd over the other REEs in a mixed system.

## 4 Conclusion

Separation of Gd(III) from Sm(III) and La(III) using TBP ligand by ELM with BBD method obtained optimum results at 4.2% surfactant concentration, 1.4% ligand concentration, 2.3 M internal aqueous phase concentration, and pH of the external aqueous phase is 1. Separation of Gd(III) from Sm(III) and La(III) using TBP by ELM with the BBD method obtained %E value of Gd(III) 84.18%, Sm(III) 64.95%, La(III) 37.19%; %S Gd(III) 89.24%, Sm(III) 70.18%, La(III) 55.91%; recoveries Gd(III) 75.12%, Sm(III) 45.58%, La(III) 20.79%; and purity Gd(III) 22.59%, Sm(III) 27.41%, La(III) 50.00%.

## Conflict of Interest

The authors declare no conflict of interest.

## Data Availability

The data that support the findings of this study are available on request from the corresponding author. The data are not publicly available due to privacy or ethical restrictions.
